# Atomic scale study of stress-induced misaligned subsurface layers in KDP crystals

**DOI:** 10.1038/s41598-019-46672-0

**Published:** 2019-07-18

**Authors:** Yue Hu, Zhen Zhu, Jiamin Xiao, Hezhu Shao, Li Zhao, Min Xu, Jun Zhuang

**Affiliations:** 1 Shanghai Ultra Precision Optical Manufacturing Engineering Technology Research Center, Department of Optical Science and Engineering, Fudan University, Shanghai, 200433 China; 2 State Key Laboratory of Surface Physics and Department of Physics, Fudan University, Shanghai, 200433 China; 3 Ningbo Institute of Materials Technology and Engineering, Chinese Academy of Sciences, Ningbo, 315201 China

**Keywords:** Electronic properties and materials, Electronic structure

## Abstract

We carried out *ab initio* calculations to study the atomic configuration, band structure and optical absorption of the lattice misalignment structure (LMS) in a subsurface layer of a machined KH_2_PO_4_ (KDP) crystal. By varying the different degrees of misalignment, the changes in the corresponding atomic position and bond and energy are obtained, and their correlations are analysed in detail. The results indicate that in the LMS evolution, the variation in the proton distribution around the oxygen atoms plays an important role, and many local stable LMSs appear. Interestingly, at a certain misalignment value, the total system energy of the local stable LMS is near that of a perfect KDP crystal. For some local stable LMSs, the electronic and optical properties related to the laser damage threshold (LDT) of KDP are further studied. The results show that in comparison with a perfect KDP crystal, the band gaps of local stable LMSs at some certain misalignment values become narrow, and their optical absorption curves produce an obvious redshift. These facts demonstrate that the emergence of the LMS could have a significant impact on the optical absorption of the KDP material and thus affect the LDT of KDP under certain working conditions.

## Introduction

Owing to the fact that KH_2_PO_4_ (KDP) crystals not only rapidly grow to large-scale optics but also have remarkable nonlinear optical and electro-optical properties^1–4^, these crystals have been widely used for light frequency converters and Pockels photoelectric switches in high-power large-aperture laser systems, such as inertial confinement fusion (ICF) reactors^5^. To date, a KDP-type crystal is still the most suitable nonlinear material that meets the requirements of various high-power large-aperture laser systems^6^. However, the laser damage threshold (LDT) of a machined KDP crystal is far below its intrinsic damage threshold^7–9^, which has become one of the key technical bottlenecks that severely hinders the development of ICF reactors. Hence, extensive studies on machining-induced damage and its effect on the properties of the KDP material have been carried out^10–14^. For example, on a micron or larger scale, various surface and subsurface defects, such as pits, cracks, and nicks, caused during KDP lens surfacing by different methods have been reported. These defects may have an impact on the distribution of the light field and thus affect the LDT of the KDP crystals. Certainly, by optimizing the machining techniques and/or parameters, relative large-scale damage can be reduced or even eliminated^15^. However, damage at a more micro-level is almost impossible to avoid, even with a very advanced surface machining technique. For example, various atomic-level defects could be produced or enhanced after machining. Recently, using grazing incidence X-ray diffraction, Hou *et al*. more precisely evaluated the subsurface structure of a KDP crystal after ultra-precision surface machining. Atomic-level damage referred to as the lattice misalignment structure (LMS) induced by mechanical stresses is observed in the shallow subsurface layer, which can be along various planes^16^. Considering the inescapability of this micro-damage and the possibility of a lower LDT, clarifying the material properties of KDP crystals becomes more important. Compared to other atomic-level damage characteristics, such as point defects^17–19^ and dislocations, which also usually appear during crystal growth, unique machining-induced defects, such as LMSs, and their effects on the optical properties and the LDT of the KDP crystals have rarely been studied.

For the first time to our knowledge, aiming at the damage of the LMS, in the present work, we carry out *ab initio* calculations to investigate the atomic configuration, band structure and optical absorption of KDP. By varying with different degrees of misalignment, the changes in the atomic position and bond and energy are obtained, and their correlations are analysed in detail. For some local stable LMSs, the structural characteristics are discussed, and the electronic band structures and optical absorption are further studied. The optical absorption is closely related to the LDT of the KDP crystals, and its magnitude is helpful for evaluating the influence of LMS damage on the LDT.

## Model and Method

According to a recent grazing incidence X-ray diffraction experiment^16^, there are multiple LMSs in the shallow subsurface layer of a machined KDP crystal due to shear or hydrostatic stresses. The misalignments are along the (112), (312), (200), (101), (301), (303) and (220) planes, among which the intensity of the (200) diffraction peak is strongest. Therefore, as a first step in the research, the LMS along the (200) plane is considered.

**Figure 1:**
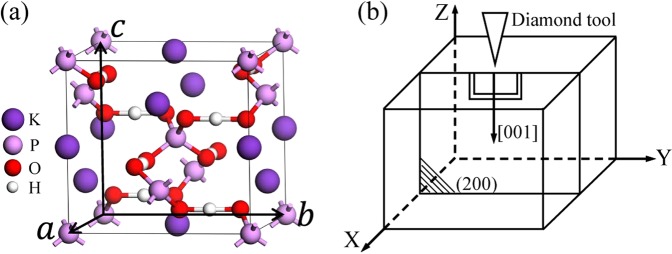
Primitive cell of KDP. (**a**) The white, red, pink and purple balls denote H, O, P and K atoms, respectively, which are all the same in the following figures. A schematic diagram of the experimental settings (**b**).

**Figure 2:**
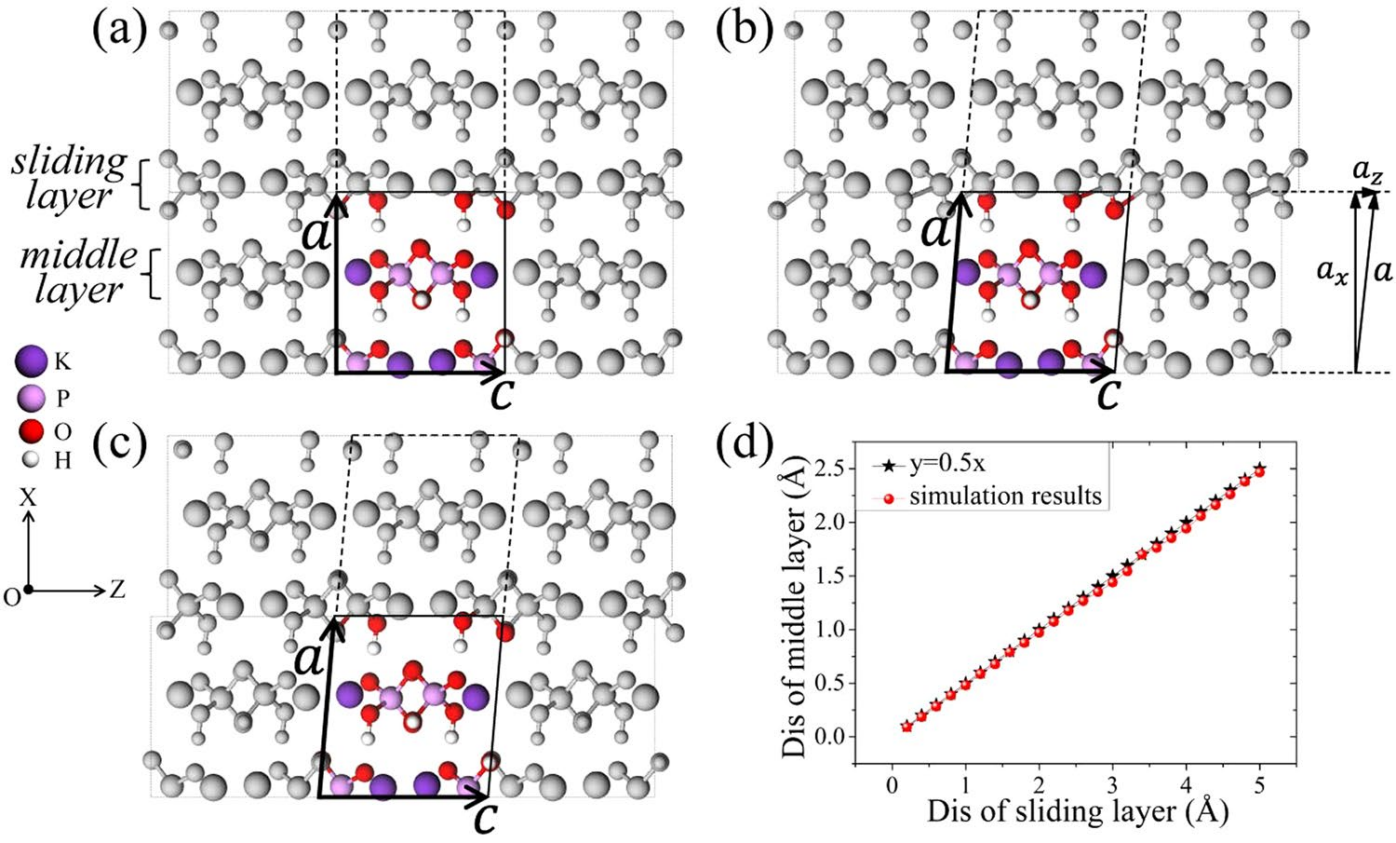
The solid-line box containing the colored atoms is the unit cell we simulated, and the grey atoms are only obtained by the periodic boundary conditions. The initial structure (**a**), by adjusting the z-component of vector ***a***, we move the grey atoms in the dashed box a displacement *d*_*z*_ (*d*_*z*_ = *a*_*z*_) in the Z direction, before (**b**) and after (**c**) relaxation. The displacement (dis) relation between the middle layer and the sliding layer (**d**).

At room temperature, KDP forms a paraelectric phase with the tetragonal ${I}\overline{4}2{d}$ space group with lattice constants *a* = *b* = 7.4 Å and *c* = 7.0 Å. There are 32 atoms in the unit cell of KDP, as shown in Fig. 1(a). By applying the periodic boundary conditions ***a*** = (*a*_*x*_, 0, 0), ***b*** = (0, *b*_*y*_, 0) and ***c*** = (0, 0, *c*_*z*_), one can simulate a perfect bulk system. As shown in Fig. 2(a), the solid-line box containing the colored atoms is the unit cell, and the dashed box containing the grey atoms is only obtained by the periodic boundary condition in the X direction. According to the literatures^13,16^, the (200) LMS is thought to be a result of the stress generated by the cutting tip, which is along the Z direction, and the corresponding force schematic diagram is given in Fig. 1(b). There are two possible ways to apply the initial stress to the system, corresponding to two kinds of (200) boundaries: one crosscuts the H-bonds and the other crosscuts the P-O bonds. For the former, the initial stress is applied to the system by artificially stretching the H-bonds on the boundary; and for the latter, the initial stress is applied to the system by artificially stretching the P-O bonds on the boundary. Then, through the structural optimization, we expect that the stress in the deformed bond can be transferred to every layer in the system, during which the (200) LMS appears. Under this expectation, we performed a compared test for these two cases. The results show that after the structural optimization, the slightly deformed H-bond is still in the stretched state, and there is one layer that hardly moves, which means that the initial stress concentrated in the deformed H-bonds is hardly transferred; however, the slightly deformed P-O bonds completely recover to the initial bond length, and meanwhile the (200) LMS appears naturally. Therefore, we only consider the case where the (200) boundary crosscuts the P-O bonds, as shown in Fig. 2(a). To simulate the formation/evolution process of the (200) LMS under the stress, the molecular statics method is used^20–23^. Specifically, first of all, by changing the periodic boundary conditions like ***a*** = (*a*_*x*_, 0, *a*_*z*_), ***b*** = (0, *b*_*y*_, 0) and ***c*** = (0, 0, *c*_*z*_), as shown in Fig. 2(b), we move the grey atoms in the dashed box along the Z direction a small displacement *d*_*z*_ (*d*_*z*_ = *a*_*z*_) relative to the colored atoms in the unit cell to model the strain induced by the force from the cutting tip. The P-O bonds at the (200) boundary of the unit cell are now slightly stretched or compressed, as seen in Fig. 2(b). In the simulation, the displacement *d*_*z*_ is small enough to keep the P-O bonds from breaking. Then, the system is fully relaxed. After relaxation, we move the grey atoms in the dashed box in the Z direction again, and repeating the above process. By this way, we try to simulate the atomic evolution of the material under the increasing z-component of the stress from the cutting tip. After relaxation at each step, we have two expectations. On one hand, in view of the strong attraction between the oxygen atoms and the phosphorous atoms, we expect that the stretched or compressed P-O bonds can recover to the original bond length through relaxation, and thus make the atoms near the top boundary of the unit cell move with a displacement *d*_*z*_ as a whole, which we call it as the sliding layer marked in Fig. 2(a). On the other hand, we expect the middle layer indicated in Fig. 2(a) also slides in the Z direction with the sliding layer in view of the attraction of H-bonds. Thus the (200) LMS observed in the experiment could appear in the simulation, in which the details of the LMS evolution and the corresponding structural characteristics can be obtained that are hard to be obtained directly from the experiment.

The *ab initio* calculations we performed are based on density function theory (DFT) utilizing the Vienna *Ab initio* simulation program pack (VASP)^24^ package. The exchange correlation function is described by the Perdew-Becke-Ernzerhof (PBE) generalized gradient approximation (GGA)^25^. Ultrasoft pseudopotentials^26^ are used for the electron-ion interactions with a 680 eV cutoff energy. A 3 × 3 × 3 Monkhorst-Pack k-point mesh is used for the case of a 32-atom unit cell. With these parameters, the perfect bulk KDP crystal is first optimized until the forces on each atom are smaller than 0.01 eV/Å, and the obtained lattice constants are *a* = *b* = 7.6 Å and *c* = 7.2 Å. The O-O and O-H bond lengths are 2.499 Å and 1.068 Å, which are in good agreement with the experimental values of 2.495 Å and 1.066 Å, respectively^27^. The optimized initial structure is shown in Fig. 2(a). Then, we move the grey atoms in the dashed box along the Z direction one step 0.2 Å by changing the z-component of vector ***a***, as shown in Fig. 2(b). It is obvious that the change in *a*_*z*_ also leads to a change in the distance in the X direction and the interaction among each layers. Therefore, *a*_*x*_ is adjusted accordingly to create the lowest energy. That is, with the fixed *a*_*z*_, *b*_*y*_ and *c*_*z*_, all atoms in the system are fully relaxed and *a*_*x*_ is adjusted simultaneously until the forces on each atom are smaller than 0.05 eV/Å. As expected, after relaxation, the stretched or compressed P-O bonds resulting from the displacement basically recover to the original length, which makes the sliding layer move one step 0.2 Å as a whole in the Z direction, as shown in Fig. 2(c). Furthermore, the middle layer, which is attracted by the sliding layer through the H-bonds, also slides in the Z direction. The displacement of the middle layer is approximately half of one step because it is affected by symmetric H-bonds in the X direction. That is, the (200) LMS with a small misalignment of 0.1 Å is formed. Repeating the above process, the displacement *d*_*z*_ then gradually increases in steps of 0.2 Å. After relaxation at each step, the displacement of the middle layer remains nearly half of that of the sliding layer, as shown in Fig. 2(d). That is, by the molecular statics method described above, the (200) LMS indeed appears in the simulation, and different displacement *d*_*z*_ means different degree of misalignment structure, which is capable of studying the details of the LMS evolution.

## Results and Discussion

**Figure 3:**
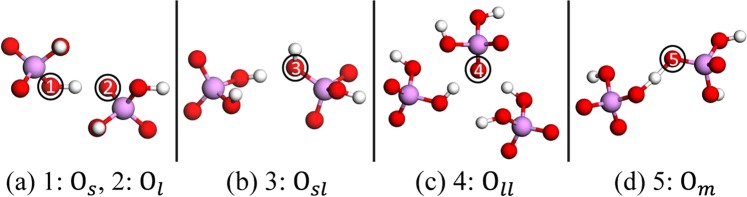
Schematic of various oxygen states that appear in the sliding process. The labelled numbers and black circles indicate five types of oxygen states: O_*s*_ and O_*l*_ (a), O_*sl*_ (b), O_*ll*_ (c) and O_*m*_ (d), meanings of which are given in the text.

With an increasing degree of misalignment, we first focus on the atomic position, bond and energy changes. It is known that for a KDP crystal, its structure can be viewed as a three-dimensional skeleton hydrogen bond system, in which PO_4_ tetrahedrons are interconnected by H-bonds. Initially, the H-bonds are in the X or Y direction, as shown in Fig. 2(a). At room temperature, KDP forms a paraelectric phase^28,29^, and the proton in the H-bond is off-centred in the two oxygen atoms^30,31^, as shown in Fig. 3(a). That is, there are two bond distances (short and long) between the related proton and oxygen atom^32^. For convenience, as shown in Fig. 3(a), the oxygen atom and the proton connecting through the short distance are expressed as “O_*s*_” and “*s*”, respectively; otherwise, they are indicated as “O_*l*_” and “*l*”, respectively. When misalignment occurs, the distances certainly change, and the proton may escape from one oxygen to another, which makes a certain oxygen atom have two protons, as shown in Fig. 3(b,c). Even if these processes occur, our simulation results show that the distances between the related proton and oxygen atom can still be classified into two types: short (0.998~1.218 Å) and long (1.277~1.848 Å). Therefore, with respect to the oxygen atom that has two protons resulting from the LMS, e.g., those in Fig. 3(b,c), we can still use symbols such as O_*sl*_ and O_*ll*_ to roughly describe the type of oxygen state, respectively. Furthermore, before a proton escapes from one oxygen to another, there might be a transitional structure at a certain misalignment, in which two bond distances (short and long) converge to a single distance (medium, 1.218~1.277 Å). In this case, as shown in Fig. 3(d), the oxygen atom and the proton connecting through the medium distance are therefore described by symbols such as “O_*m*_” and “*m*”, respectively.

**Figure 4:**
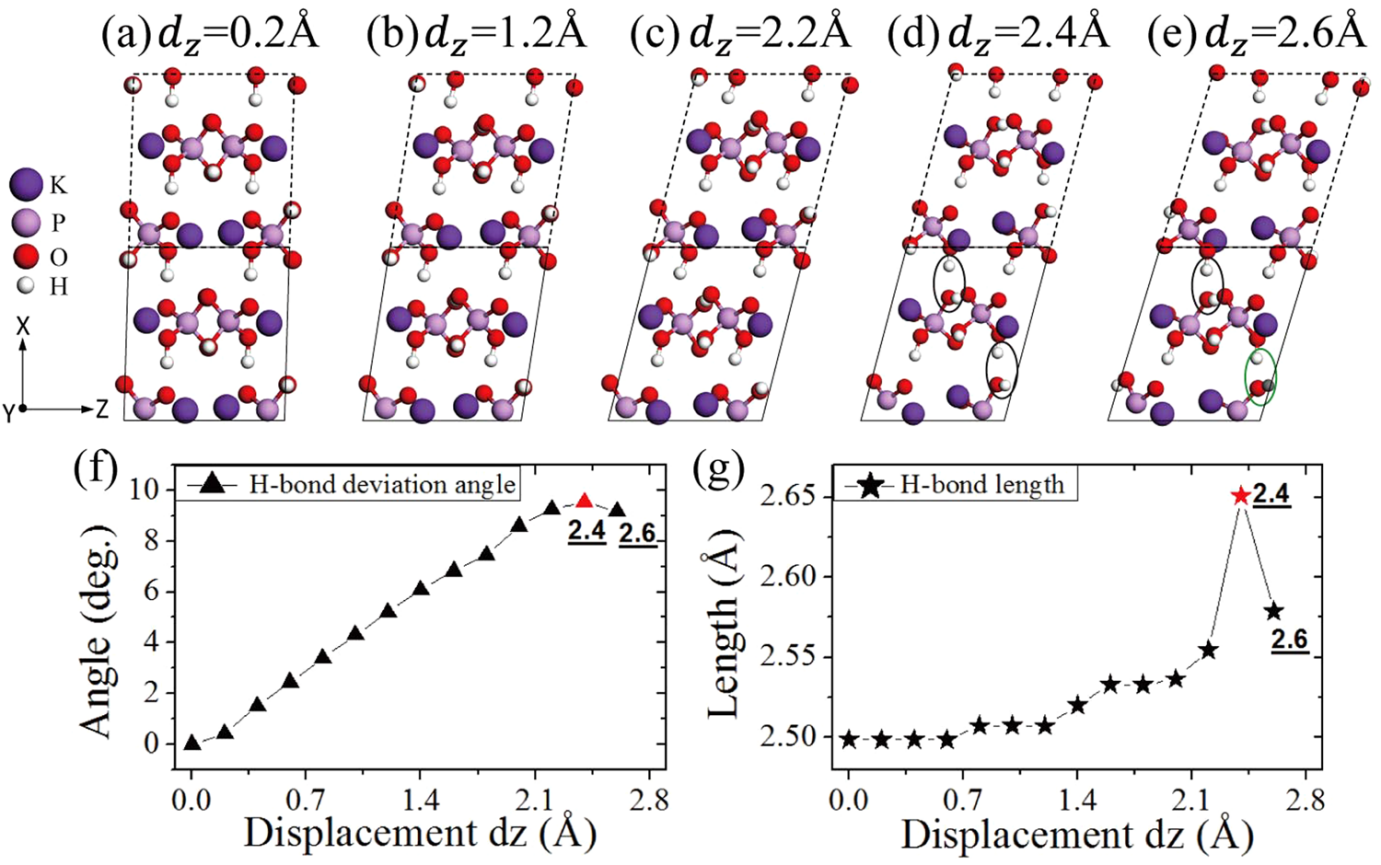
Misalignment structures at 0.2 Å (**a**), 1.2 Å (**b**), 2.2 Å (**c**), 2.4 Å (**d**) and 2.6 Å (**e**). The labelled black ellipses (**d**) and green ellipses (**e**) denote the different types of oxygen states: O_*sl*_ and O_*ll*_, respectively. For clarity, the atoms in the dashed box obtained by the periodic boundary condition in the X direction are restored to colour, which is all the same in the following figures. The variation in average H-bond deviation angle (**f**) and H-bond length (**g**) with displacement *d*_*z*_.

**Figure 5:**
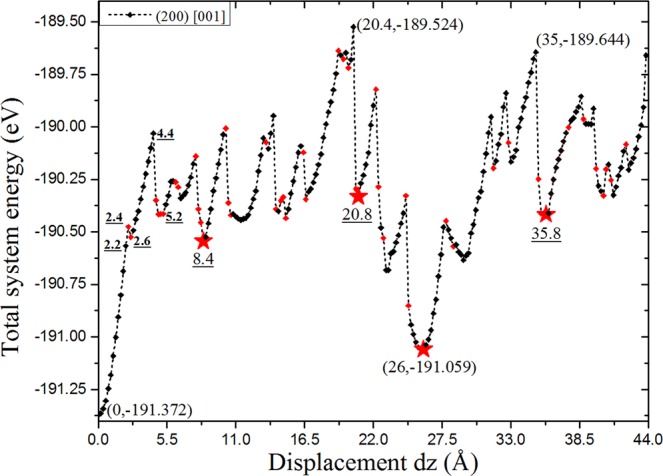
Variation in the total system energy with displacement *d*_*z*_. Many energy wells or local stable LMSs appear.

Based on our simulation results at different degrees of misalignment, we find that the structural evolution and subsequent energy changes can be mainly described and explained through the variation in oxygen states. From the start, the distribution of protons around oxygen atoms varying with different displacement *d*_*z*_ values is presented in Table 1. As clearly seen in Table 1, each PO_4_ tetrahedron in the initial structure contains two and only two types of oxygen states that are O_*s*_ and O_*l*_, respectively, which remain unchanged until the displacement *d*_*z*_ reaches 2.2 Å. Although, for a small misalignment, the oxygen states of the LMS remain the same as that of a perfect KDP crystal, the LMS produces an obvious deformation relative to the perfect KDP structure, as shown in Fig. 4(a,c), in which the H-bonds are no longer exactly in the X or Y direction. Specifically, H-bonds tilt and elongate gradually with a sliding of the (200) planes. As shown in Fig. 4(f,g), before *d*_*z*_ = 2.4 Å, the average tilt angle of the H-bond relative to its original direction gradually increases, and its average bond length also increases. In addition, the tilt and elongation of the H-bonds make the PO_4_ tetrahedrons produce a certain rotation. In short, the LMS relative to the perfect KDP structure gradually deforms with increasing displacement *d*_*z*_. This effect leads to an increase in the total system energy, as seen in the first segment (0–2.2 Å) of the energy curve in Fig. 5, which indicates that the stress in LMS is being accumulated in this displacement range. Next, if we continue to move the (200) planes along the Z direction with a displacement *d*_*z*_ of 2.4 Å, as shown in Fig. 4(d), then the change in the distribution of protons occurs; that is, two new oxygen states (O_*sl*_) appear in the LMS. The newly formed H-bond in the X direction, marked by black ellipse in Fig. 4(d), is significantly elongated. The relative average H-bond length in Fig. 4(g) therefore increases accordingly. At this step, the distribution of protons is no longer homogeneous, and the corresponding total system energy increases (see Fig. 5). If the (200) planes move one step in the Z direction (i.e., *d*_*z*_ = 2.6 Å), as shown in Fig. 4(e), then one of two oxygen states O_*sl*_ transforms into O_*ll*_. The elongated H-bonds in the previous step obviously become shorter. Therefore, the average H-bond length at this step decreases, as shown in Fig. 4(g). In addition, the average tilt angle of the H-bond relative to its original direction also slightly decreases, as shown in Fig. 4(f). The corresponding total system energy decreases (see Fig. 5), which suggests that the oxygen state O_*ll*_ is more stable than O_*sl*,_ as expected. We then continue to move the (200) planes forward, and the distribution of protons around oxygen atoms remains unchanged until 4.4 Å, as seen in Table 1. This behaviour means that the variation in the LMS in this segment is just deformation, which is similar to that in the first segment of the energy curve range from 0 Å to 2.2 Å. After 4.4 Å, as shown in Table 1, the distribution of protons changes again until 5.2 Å, as in the range of 2.4 Å to 2.6 Å. In the following sliding process, the structural evolution basically repeats in the above way. The corresponding total system energy curve is given in Fig. 5. The figure shows that, as mentioned above, the total system energy initially increases with increasing displacement *d*_*z*_ from 0 Å to 2.2 Å, but an abrupt decrease occurs at a certain displacement of 2.6 Å, and this variation also repeats with sliding. For a clear presentation, as shown in Fig. 5, we use red solid rectangles to mark the energy curve when the distribution of protons or the type of oxygen states changes. It can be clearly seen that whenever the distribution of protons or the type of oxygen states changes, near or after this structure, the total system energy is usually accompanied by a sudden change; otherwise, it only gradually increases as a result of structural deformations. The above facts give us a clear LMS evolution picture; specifically, when misalignment occurs, the system first releases part of the stress through structural deformations, such as PO_4_ tetrahedron rotation and H-bond tilting and elongation. However, due to the limited effect of such a deformation, the stress and energy will still accumulate and increase as the degree of misalignment increases. When these deformations accumulate and increase to a certain extent, a qualitative change occurs; that is, the distribution of protons around the oxygen atoms or the type of oxygen states changes. Thereafter, the total system energy usually significantly decreases and eventually forms a local stable LMS. Afterwards, if the degree of misalignment continues to increase, then, starting with this local stable LMS, the above process is repeated.

**Table 1:** Variation in the proton distribution around oxygen atoms with displacement *d*_*z*_.

*d*_*z*_ (Å)	1_a_	1_b_	1_c_	1_d_	2_a_	2_b_	2_c_	2_d_	3_a_	3_b_	3_c_	3_d_	4_a_	4_b_	4_c_	4_d_
0–2.2	*s*	*s*	*l*	*l*	*l*	*s*	*l*	*s*	*s*	*s*	*l*	*l*	*l*	*s*	*l*	*s*
2.4	*s*	*s*	*l*	*l*	*l*	*s*		*sl*	*s*	*s*	*l*	*l*	*l*	*s*		*sl*
2.6–4.4	*s*	*l*	*s*	*l*	*s*	*s*		*ll*	*s*	*s*	*l*	*l*	*l*	*s*		*sl*
4.6	*s*	*l*	*s*	*l*	*s*	*s*		*ll*	*s*	*s*	*l*		*l*	*s*	*l*	*sl*
4.8	*s*	*l*	*s*	*s*	*s*	*s*		*ll*	*s*	*l*	*s*		*s*	*l*	*l*	*ll*
5	*s*	*l*	*s*	*m*	*s*	*s*		*ll*	*s*	*l*	*s*		*s*	*m*	*l*	*ll*
5.2–6	*s*	*l*	*s*	*l*	*s*	*s*		*ll*	*s*	*l*	*s*		*s*	*s*	*l*	*ll*
8.4–10	*s*	*s*	*ll*		*ll*	*s*		*s*	*s*	*s*	*s*		*l*	*s*	*l*	*ll*
20.6–22	*l*	*s*	*s*			*s*	*ll*	*s*	*s*	*s*	*ll*		*l*	*ll*	*s*	*s*
24.8–27.6	*l*	*s*	*l*	*s*	*l*	*s*	*l*	*s*	*s*	*l*	*l*	*s*	*l*	*l*	*s*	*s*
35.4–37.2	*s*	*ll*		*s*	*sl*	*l*		*s*	*s*		*l*	*s*	*ll*	*l*	*s*	*s*

In the first row, the Arabic numerals (1, 2, 3, 4) are the series numbers of four PO_4_ tetrahedrons in the primitive cell of KDP, and the subscripts a, b, c and d denote four oxygen atoms in each PO_4_ tetrahedron, respectively.

**Figure 6:**
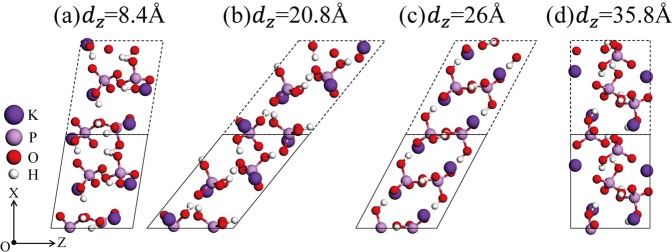
Four local stable LMSs at 8.40 Å (**a**), 20.8 Å (**b**), 26.0 Å (**c**) and 35.8 Å (**d**).

Many energy wells in Fig. 5 indicate that various local stable LMSs appear in the sliding process, among which the LMS at 26 Å is very interesting because its total system energy is approximately 0.3 eV higher than that of a perfect KDP crystal. Here, we select four typical local stable LMSs at 8.40 Å, 20.8 Å, 26.0 Å and 35.8 Å, corresponding to four energy wells that are marked by red stars in Fig. 5. As clearly seen in Table 1, in comparison with the initial structure, the proton distribution around oxygen atoms in the LMSs at 8.40 Å, 20.8 Å and 35.8 Å are no longer homogeneous. Specifically, for LMSs at 8.40 Å and 20.8 Å, the same types of new oxygen states that are 3O_*ll*_ appear in the primitive cell, but for the LMS at 35.8 Å, different types of new oxygen states occur in the primitive cell: one is O_*sl*_ and the other two are 2O_*ll*_. In contrast to these three local stable LMSs, the distribution of protons around oxygen atoms in the LMS at 26 Å is still homogenous; that is, each PO_4_ tetrahedron only contains two types of oxygen states, namely, O_*s*_ and O_*l*_, which are fully identical to those in a perfect KDP crystal. Furthermore, in addition to the change in the type of oxygen states, as shown in Fig. 6(a,d), the local stable LMSs produce a considerable deformation relative to the perfect KDP structure in Fig. 2(a). The H-bond deviates significantly from its original direction, and the PO_4_ tetrahedron produces an obvious rotation relative to its initial position. Nevertheless, the structural deformation of the LMS at 26 Å, as shown in Fig. 6(c), still has an obvious difference compared to that of the LMSs at 8.40 Å, 20.8 Å and 35.8 Å in Fig. 6(a,b,d), respectively. For the former, its structure can be approximately viewed as having an overall tilt relative to the initial structure, in which the H-bonds are parallel to each other, but for the latter ones, the structures are apparently configurationally disordered, in which the H-bonds are in different directions. These results imply that the LMS at 26 Å would be relatively stable; as expected, its total system energy is significantly lower than that of other local stable LMSs (see Fig. 5). In summary, with a sliding of the (200) planes, many local stable LMSs can appear, and their structures change considerably relative to the perfect KDP structure. However, it is interesting to see that among these structures, there is still a special LMS, in which the oxygen states remain unchanged and its total system energy can be compared to a perfect KDP crystal. Furthermore, in view of the existence of various stresses in the actual machining process, all these local stable LMSs have the possibility of appearing in the material; thus, their electronic and optical properties deserve further study.

**Figure 7:**
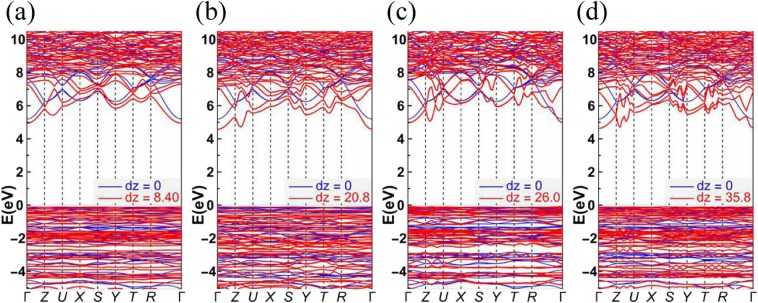
Band structures of a perfect KDP crystal (blue lines) versus the local stable LMSs (red lines) at 8.40 Å (**a**), 20.8 Å (**b**), 26.0 Å (**c**) and 35.8 Å (**c**).

**Figure 8:**
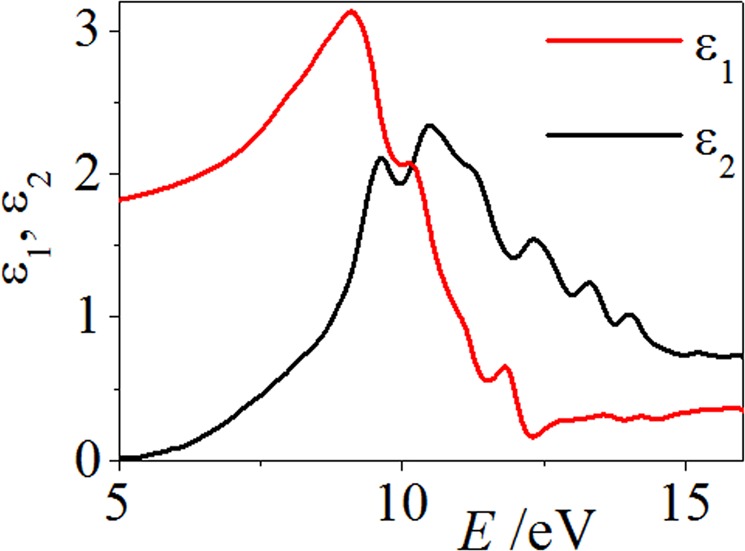
Complex dielectric functions of a perfect KDP crystal (incidence plane *xy*).

**Figure 9:**
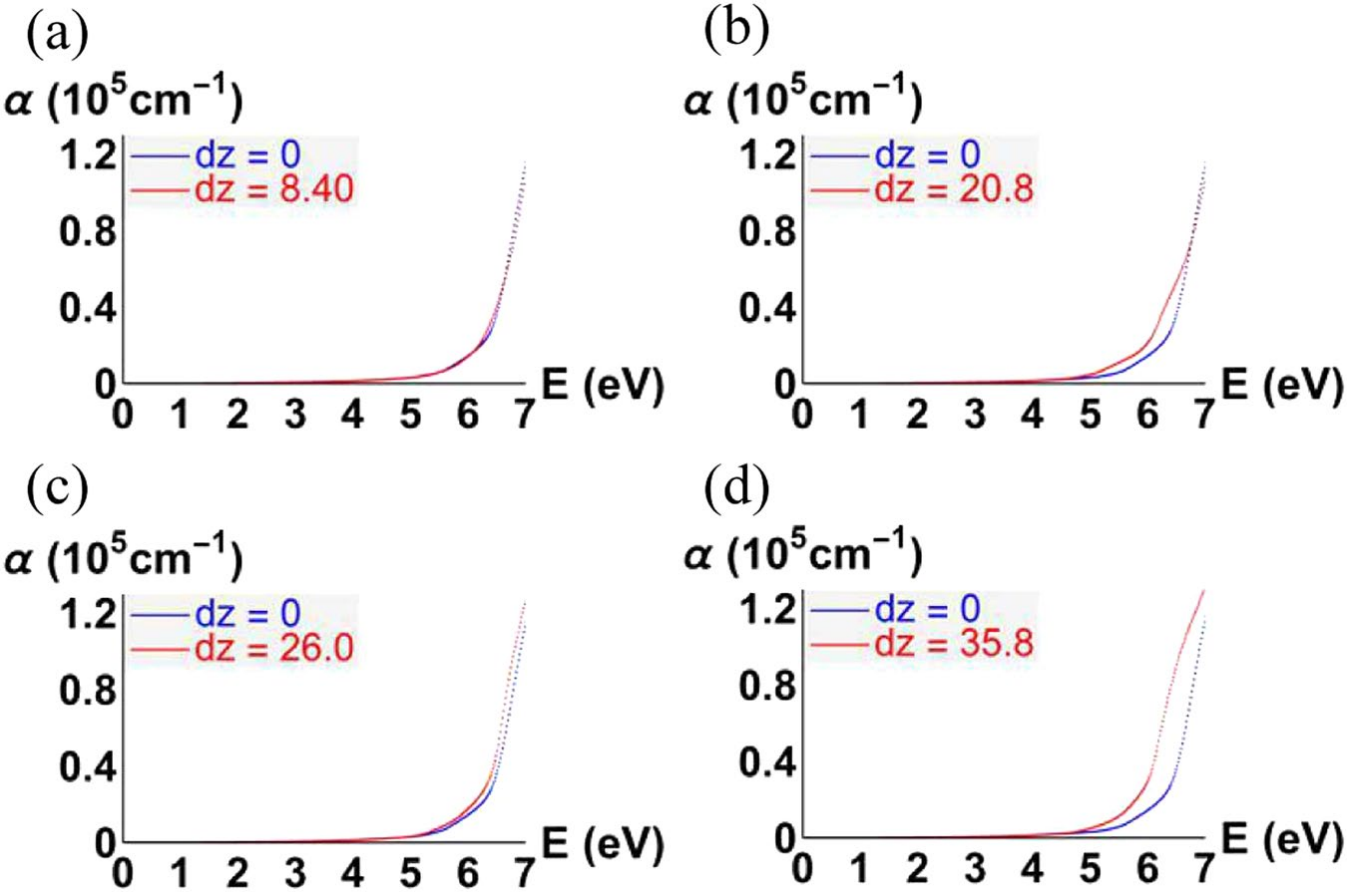
Optical absorption of a perfect KDP crystal (blue line) versus the local stable LMSs (red line) at 8.40 Å (**a**), 20.8 Å (**b**), 26.0 Å(**c**) and 35.8 Å (**c**) (incidence plane *xy*).

As shown in Fig. 7, we give the comparative band structures of a perfect KDP crystal and the local stable LMS to evaluate the influence of LMS damage on the LDT of the KDP crystals. Their band structures have been found to be very different, among which the band structure of the LMS at 26 Å is closer to that of a perfect KDP crystal. As shown in Fig. 7(c), the band structure in the energy range from −2.4 eV to −3 eV and from −3.8 eV to −4 eV are energy separated, which is similar to that of a perfect KDP crystal. However, for the other local stable LMSs, as shown in Fig. 7(a,b,d), three groups of bands in the energy range of −5~0 eV are almost energy connected. This energy connection suggests that great delocalization occurs in the corresponding electronic states, which can be mainly attributed to a variation in the distribution of protons around the oxygen atoms. Furthermore, from Fig. 7, the band gaps of local stable LMSs become narrow relative to that of a perfect KDP crystal, especially for the LMSs at 20.8 Å and 35.8 Å. Specifically, the obtained band gap of a perfect KDP crystal is 5.3 eV, which is in good agreement with the previous theoretical value of 5.06 eV^33^. However, for the LMSs at 8.40 Å, 20.8 Å, 26.0 Å and 35.8 Å, the band gaps are 5.1 eV, 4.7 eV, 5.1 eV and 4.7 eV, respectively. It is well known that the intrinsic deficiency of the DFT underestimates the band gap. In view of the experiment value of 7.3 eV^34^ and applying a scissor operator for a correction, we obtain the corrected band gaps of the LMSs at 8.40 Å, 20.8 Å, 26.0 Å and 35.8 Å, which are 7.1 eV, 6.7 eV, 7.1 eV and 6.7 eV, respectively. Furthermore, we also provide the comparative optical absorption curves of a perfect KDP crystal and the local stable LMS, which are obtained from the function of $I({\rm{\omega }})=\sqrt{2}(\omega ){(\sqrt{{\varepsilon }_{1}{(\omega )}^{2}+{\varepsilon }_{2}{(\omega )}^{2}}-{\varepsilon }_{1}(\omega ))}^{1/2},$ where *ε*_1_ and *ε*_2_ are the real part and imaginary part of the dielectric function, respectively. The imaginary part of the dielectric function is obtained by summing over independent transitions between Kohn-Sham states neglecting local field effects^35^, and the real part is calculated from the imaginary part by using the Kramers-Kroning relations. As a benchmark, the *ε*_1_ and *ε*_2_ for the perfect KDP crystal (*d*_*z*_ = 0 Å) are given in Fig. 8, which are in agreement with the literature^36^. As expected, the optical absorption curve of the LMSs at 20.8 Å and 35.8 Å produced an obvious redshift relative to that of a perfect KDP crystal (see Fig. 9). These results demonstrate that the electronic and optical properties of the KDP material have been conspicuously altered by the LMSs, such as those structures at 20.8 Å and 35.8 Å. These structures may be one part of the initially absorbing sites in the surface/subsurface of a machined KDP crystal and may thus affect the output power of a high-energy laser system under certain working conditions^34,37^. Furthermore, the LMSs at 20.8 Å and 35.8 Å are just two examples, and more similar local stable LMSs may appear in view of the existence of various stresses during the actual machining process.

## Conclusions

We carried out *ab initio* calculations to study the atomic configuration, band structure and optical absorption of the LMS in a subsurface layer of a machined KDP crystal. With a sliding of the (200) planes, the atomic position and bond and energy changes for different LMSs are obtained, which gives us a clear LMS evolution picture; that is, the system releases part of the stress initially through structural deformations with an increasing degree of misalignment, such as PO_4_ tetrahedron rotation, H-bond tilting and elongation. However, due to the limited effect of such deformations, the stress and energy will still accumulate and increase as the degree of misalignment increases. When the misalignment accumulates and increases to a certain extent, a qualitative change occurs; that is, the distribution of protons changes; thereafter, the total system energy usually significantly decreases and eventually forms a local stable LMS. As expected, with an increase in the degree of misalignment, various local stable LMSs can appear. For most of them, both the structural details and the distribution of protons or oxygen states produce significant changes in comparison with a perfect KDP crystal. Interestingly, there is still a special LMS in which the distribution of protons or oxygen states remains unchanged and its total system energy can be compared to a perfect KDP crystal. Moreover, for four typical LMSs, we further studied their electronic and optical properties related to the LDT of the KDP crystals. The results show that in comparison with a perfect KDP crystal, the band gaps of the local stable LMSs at 20.8 Å and 35.8 Å become narrow, and their optical absorption curves produce an obvious redshift. The facts demonstrate that the emergence of the LMS does have a significant impact on the optical absorption of the KDP material, which suggests that under certain working conditions, the LDT of the KDP crystals will be very much affected by such machining-induced damage.
